# Statuserhebung der österreichischen Schmerzambulanzen 2023: Einfluss der COVID-19-Pandemie auf die schmerzmedizinische Versorgung

**DOI:** 10.1007/s00482-025-00871-z

**Published:** 2025-03-17

**Authors:** Sascha Hammer, Anna Krawczyk, Brigitte Messerer, Stefan Neuwersch-Sommeregger, Patrick Reinbacher, Gregor Schittek, Andreas Sandner-Kiesling

**Affiliations:** 1 Klin. Abt. für Anästhesiologie und Intensivmedizin I, Universitätsklinik für Anästhesiologie und Intensivmedizin, Medizinische Universität Graz, Auenbruggerplatz 5/5, 8010 Graz, Österreich; 2 Universitätsklinik für Orthopädie und Traumatologie, Medizinische Universität Graz, Graz, Österreich; 3 Abteilung für Anästhesiologie und Intensivmedizin, Klinikum Klagenfurt am Wörthersee, Klagenfurt am Wörthersee, Österreich

**Keywords:** Betriebszeiten, Chronischer Schmerz, Versorgungsdefizit, Multimodale Schmerztherapie, Schließungen, Operating hours, Chronic pain, Care deficits, Multimodal pain therapy, Closing of pain clinics

## Abstract

**Hintergrund:**

Laut dem Bericht der Österreichischen Schmerzgesellschaft von 2020 leiden bis zu 1,8 Mio. Menschen in Österreich unter chronischem Schmerz. Die Behandlung dieser SchmerzpatientInnen sollte laut ÖSG idealerweise mittels multimodaler Schmerztherapie in speziellen schmerztherapeutischen Einrichtungen erfolgen. Im Rahmen der vorliegenden Arbeit soll der postpandemische Ist-Versorgungszustand 2022 der österreichischen Schmerzambulanzen erhoben und dessen Veränderung in den letzten Jahren aufgezeigt werden.

**Methoden:**

Bei dieser Befragung wurden die ärztlichen LeiterInnen aller anästhesiologischen Abteilungen Österreichs über einen Zugangscode zum Vergleich des prä- zu postpandemischen Status ihrer Schmerzambulanzen via SurveyMonkey (SurveyMonkey Inc., San Mateo, CA, USA) befragt. Es wurden nur anästhesiologische Abteilungen kontaktiert, da Schmerzambulanzen in Österreich fast ausschließlich anästhesiologisch geführt sind. Diese Umfrage wurde online per E‑Mail an alle österreichischen Kliniken mit einer Schmerzambulanz verschickt und war im Zeitraum von Herbst 2022 bis Mai 2023 zur Bearbeitung freigegeben.

**Ergebnisse:**

92 der 109 befragten Kliniken nahmen an der Umfrage teil. Davon betreiben aktuell 51 eine Schmerzambulanz, sieben im Vollzeitbetrieb. Neun Schmerzambulanzen wurden seit 2014 geschlossen, sieben neue geöffnet. Trotzdem gingen im Vergleich zu 2014 7,5 % der aktiven Schmerzambulanzen verloren. Eine gezielte Frage nach der Durchführung einer standardisierten multimodalen Schmerztherapie beantworten nur ein Schwerpunktkrankenhaus und zwei Krankenhäuser der Basisversorgung positiv. Invasive Verfahren werden häufiger angeboten als die multimodale Therapie.

**Diskussion:**

Bei einem relativen Verlust trotz nominellen Zuwachses an Schmerzambulanzen besteht eine mehrschichtige Diskrepanz zwischen der Empfehlung der Österr. Schmerzgesellschaft und der Versorgungsrealität von chronischen SchmerzpatientInnen in Österreich. Es gilt, administrative und personelle Hindernisse zu überwinden, gleichzeitig das Interesse am Management chronischer SchmerzpatientInnen wieder unter der Kollegenschaft zu wecken, um diese PatientInnengruppe in Zukunft besser zu versorgen. Eine stärkere Unterstützung seitens der Gesundheitspolitik und eine wirkungsvollere Integration von Schmerzambulanzen in das Gesundheitssystem könnten dazu beitragen, die Versorgungssituation zu verbessern.

**Graphic abstract:**

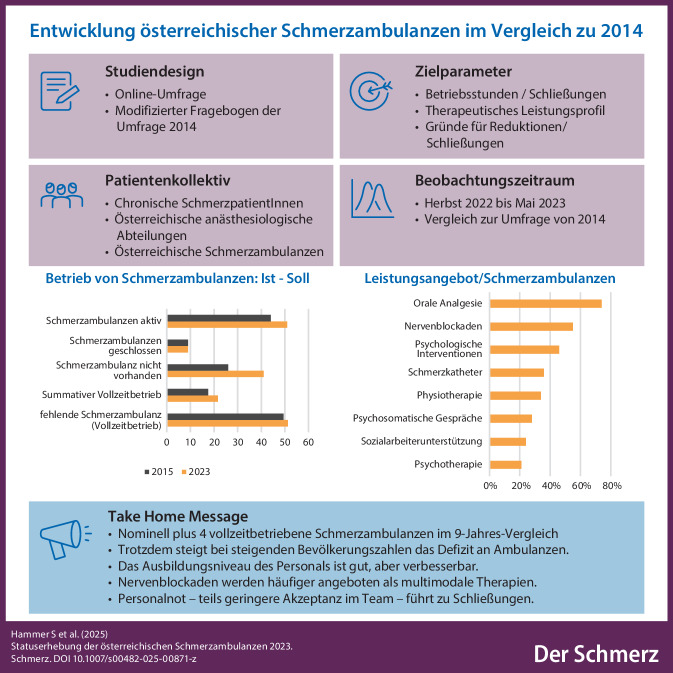

## Abstract (en)

**Background:**

According to a 2020 report by the Austrian Pain Society (ÖSG), between 1.5 and 1.8 million people in Austria suffer from chronic pain. For the treatment of these patients, multimodal pain therapy in specialized pain therapy facilities should be considered. This study aimed to analyze the actual postpandemic state of care in pain clinics in 2022 and to show how it has changed in recent years.

**Methods:**

In our survey, the heads or employees of all anesthesiology departments in Austria were asked via SurveyMonkey (SurveyMonkey Inc., San Mateo, CA, USA) using an access code to compare the pre- to postpandemic status of their pain clinics. Only anesthesiology departments were contacted, as pain clinics in Austria are almost entirely run by them. This survey was sent online by email to the heads of Austrian hospitals with an existing pain clinic and was open for completion from autumn 2022 to May 2023.

**Results:**

Of the 109 surveyed clinics, 92 participated in our study. Only 51 currently operate a pain clinic, seven of which are full-time. Nine pain clinics had closed since 2014, while seven new ones have opened. According to our data, only one specialized hospital and two basic care hospitals offer multimodal pain therapy. Invasive procedures were used more often compared to multimodal pain therapy.

**Conclusion:**

Despite the nominal increase of pain clinics in Austria, there is a multifactorial discrepancy between the recommendation of the Austrian Pain Society and the actual care of patients with chronic pain in Austria. Administrative and personnel obstacles must be overcome to meet the needs of this patient group. In addition, efforts to increase the interest of physicians in chronic pain management are needed. Greater support from healthcare policies and effective integration of pain clinics into the healthcare system could help improve the care situation.

## Einleitung

Chronischer Schmerz ist eines der häufigsten Gesundheitsprobleme der heutigen Zeit [23]. Laut einem Bericht der Österreichischen Schmerzgesellschaft (ÖSG) von 2020 leiden zwischen 1,5 und 1,8 Mio. Menschen in Österreich unter chronischen Schmerz, also bis zu ca. 20 % der Bevölkerung, und die Tendenz ist steigend [19]. Diese Anzahl findet sich in internationaler Literatur wieder [1, 4]. Chronische Schmerzen sind definiert als Schmerzen mit einer Intensität in der oberen Hälfte der Schmerzskala und länger als 3 Monate bestehend [17]. Studien belegten in diesem Falle die Wirksamkeit eines multimodalen Behandlungskonzepts zur Reduktion der Schmerzen bzw. Wiederherstellung der Funktionalität der Betroffenen [2, 14, 18]. Gerade diese modernen Konzepte ermöglichen erst die Rückkehr in den Arbeitsprozess, reduzieren die Krankenstandstage und Frühpensionsanträge. Hier reduziert der Einsatz von Mitteln (z. B. Zeit, Personal, Räumlichkeiten) für den Betrieb von medizinischen Arbeitsplätzen, die eine leitlinienbasierte multimodale Diagnostik und Behandlung durchführen, immense gesundheitsökonomische Folgekosten [5]. Da sich diese Folgekosten in Österreich auf drei unterschiedliche Geldgeber verteilen (Krankenkassen, Spitäler, Pensionsversicherung), fehlt ein gemeinsames paritätisches Bekenntnis zur Finanzierung einer multimodalen Schmerztherapie [13].

In Österreich findet daher derzeit die Behandlung chronischer Schmerzen überwiegend in Krankenhäusern mit eigens dafür eingerichteten Schmerzambulanzen statt. Diese sind nahezu ausschließlich unter anästhesiologischer Leitung. Vereinzelt werden selbstzahlende PatientInnen in Schmerzpraxen im niedergelassenen Bereich therapiert [21]. Eine multimodale schmerztagesklinische Behandlung wird nur im Klinikum Klagenfurt am Wörthersee und in Wien angeboten. In Ermangelung von Alternativen werden chronische SchmerzpatientInnen auf monodisziplinären Stationen aufgenommen, die jedoch nicht für ein leitlinienbasiertes multimodales Schmerzbehandlungskonzept vorbereitet sind. Dies protrahiert die chronischen Schmerzen, führt zur fortgesetzten Inanspruchnahme des Gesundheitssystems, und frustriert nachhaltig die betroffenen PatientInnen und das begleitende medizinische Personal [12].

Nach Jahren der schmerzmedizinischen Aufbruchsstimmung führte die Umsetzung der EU-Richtlinie 2003/88/EG zu einer Kürzung der Arbeitszeit, häufig auf Kosten des Schmerzambulanzbetriebs. 2015 existierten ca. 44 dieser speziellen Schmerzambulanzen in Österreich, wobei es zu 9 Schließungen in den 3 Jahren davor kam. Selbst damals wären mehr als doppelt so viele Schmerzambulanzen notwendig gewesen, um eine adäquate Versorgung der oben beschriebenen Anzahl an österreichischen Schmerzgeplagten zu ermöglichen [3, 12, 13]. Damit zeigte sich schon präpandemisch (vor der COVID-19-Pandemie) ein Trend zur Reduktion der Betriebszeiten und Leistungen der Schmerzambulanzen [21].

Der zweite Einschnitt erfolgte ab dem Jahr 2020: Aufgrund der COVID-19-Pandemie kam es in Österreich zu längeren Wartezeiten, zu Terminabsagen und Verschiebungen. Es mussten neue Strategien für den Ambulanzbetrieb entwickelt und etabliert werden [16].

Zum jetzigen Zeitpunkt liegen keine Daten über die postpandemische Situation der Schmerzambulanzen in Österreich vor. Daher ist das Ziel dieser Studie [23], den aktuellen Stand an Betrieb oder Schließungen der österreichischen Schmerzambulanzen zu erfassen [21], die interprofessionelle Besetzung der Schmerzambulanzen, ihre Behandlungsangebote zu erheben und um Rückmeldungen zu den beiden vorherigen Themen zu bitten [13].

## Material und Methoden

Bei dieser Befragung wurden die LeiterInnen oder Mitarbeitenden aller anästhesiologischen Abteilungen Österreichs über einen Zugangscode zum Vergleich des prä- zu postpandemischen Status ihrer Schmerzambulanzen via SurveyMonkey (SurveyMonkey Inc., San Mateo, CA, USA) befragt. Dazu wurde unser Fragebogen von der 2014/15-Befragung adaptiert und erweitert (Details siehe Anhang). Der erste Teil widmet sich der chronischen Schmerztherapie, die das Hauptthema dieser Publikation ist. Der zweite Teil fokussiert sich auf die Akutschmerztherapie. Diese Daten werden in einer Nachfolgepublikation präsentiert.

Für diese Statuserhebung wurden die österreichischen Krankenhäuser in Universitätskliniken, Schwerpunktkrankenhäuser, Krankenhäuser der Basisversorgung, Privatkliniken/Sanatorien und sonstige Krankenhäuser unterteilt. Zu den sonstigen Krankenhäusern zählen Unfallkrankenhäuser und Ordensspitäler.

Wie in der 2014/15-Umfrage fokussieren unsere zwei Hauptfragestellungen auf Existenz und Betrieb sowie auf die Gründe der Reduktionen bzw. Schließungen der österreichischen Schmerzambulanzen. Als Nebenfragestellungen erhoben wir die personellen Besetzungen bzw. Therapieangebote der Schmerzambulanzen und die Rückmeldungen der befragten ExpertInnen.

## Statistik

Die statistischen Analysen wurden mittels der Software-Programme IBM-SPSS 27 (IBM Deutschland GmbH, 71032 Böblingen, Deutschland) und Microsoft Excel 365 (Microsoft, Redmont, WA, USA) durchgeführt. Die erhobenen Daten wurden deskriptiv ausgewertet, um explorative Informationen zu gewinnen.

Ein direkter Vergleich der aktuellen Daten mit den Daten der Statuserhebung 2014/15 war nicht möglich, da der alte Fragebogen zur Präzisierung der Fragen semantisch und inhaltlich überarbeitet wurde. Wir konnten daher die Entwicklung der letzten neun Jahre nur in der Diskussion aufarbeiten.

Parallel zu unserer Publikation von 2015 ermittelten wir aus den folgenden Literaturquellen den Vergleich zwischen der Ist- und Sollschätzung österreichischer Schmerzambulanzen pro Bundesland.

- Strukturelle Mindestvoraussetzungen einer Schmerzambulanz [15]
- Bevölkerungsdichte der österreichischen Bundesländer (Statistik Austria; https://www.statistik.at/statistiken/bevoelkerung-und-soziales/bevoelkerung/bevoelkerungsstand/bevoelkerung-zu-jahres-/-quartalsanfang)
- Häufigkeit chronisch schmerzerkrankter Patienten in Österreich [4, 7]
- Wöchentliche Betriebszeiten österreichischer Schmerzambulanzen [7]

## Ergebnisse

Die Umfrage wurde im Herbst 2022 gestartet und im Mai 2023 abgeschlossen. In diesem Zeitraum wurden die Einladungen dreimal ausgesendet. Es antworteten 92 VertreterInnen der 109 kontaktierten Kliniken (Rücklaufquote 84 %).

### Existenz von Schmerzambulanzen

Von den 92 teilnehmenden Kliniken betreiben aktuell 51 (55 %) eine Schmerzambulanz. Betrachtet man die Öffnungszeiten der aktiven Schmerzambulanzen, bieten nur 7 von 51 (14 %) einen Vollzeitbetrieb mit 40 Wochenstunden an. Zwei Schwerpunktkrankenhäuser berichteten von einer Steigerung der Öffnungszeiten ihrer Schmerzambulanzen (siehe Tab. 1).

**Tab. 1:** Überblick über aktuelle Anzahl, Verteilung, Betrieb und Schließungen von Schmerzambulanzen verschiedener österreichischer Krankenhaustypen zum Zeitpunkt der Befragung (2022/23)

	Universitätskliniken	Schwerpunktkrankenhäuser	Krankenhaus der Basisversorgung	Privatklinik/Sanatorium	^*^Sonstige Krankenhäuser
Anzahl und Verteilung der beantworteten Fragebögen	6	36	39	3	8
Aktive Schmerzambulanzen	6	26	14	2	3
Vollzeitbetriebene Schmerzambulanz	5	2	–	–	–
Erhöhung der Öffnungszeiten in den letzten drei Jahren	–	2	–	–	–
Schließungen von Schmerzambulanzen	–	5	3	–	1

^*^Sonstige Krankenhäuser stehen synonym für Unfallkrankenhäuser oder Ordensspitäler

### Verteilung der Schmerzambulanzen

Von 51 aktiven Schmerzambulanzen bietet Wien mit zwölf aktiven Schmerzambulanzen den höchsten Anteil, gefolgt von der Steiermark (*n* = 9), Oberösterreich (*n* = 8), Niederösterreich (*n* = 6), Tirol (*n* = 5), Salzburg (*n* = 3). Vorarlberg und Burgenland weisen nur zwei aktive Schmerzambulanzen auf.

**Abb. 1:**
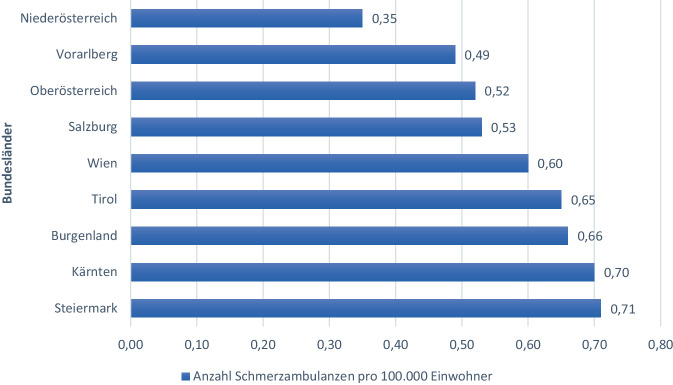
Verteilung der aktiven Schmerzambulanzen pro 100.000 Einwohner auf die österreichischen Bundesländer zum Zeitpunkt der Befragung

Die meisten aktiven Schmerzambulanzen pro 100.000 Einwohner verzeichnet die Steiermark mit 0,71 (siehe Abb. 1).

### Betrieb und Betriebszeiten der Schmerzambulanzen

**Abb. 2:**
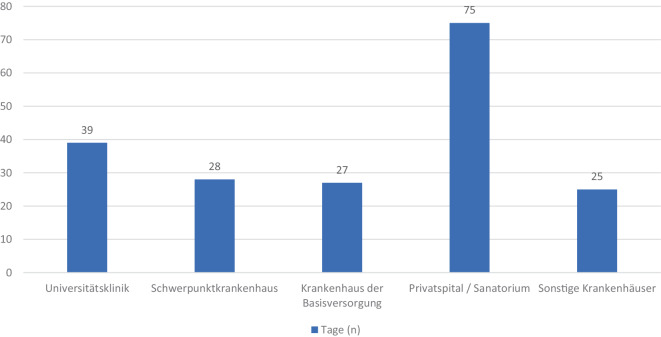
Durchschnittliche Wartezeit in Tagen von chron. SchmerzpatientInnen für ein Erstgespräch in österreichischen Schmerzambulanzen

Bei der durchschnittlichen Wartezeit in Tagen zeigen sich zwischen Krankenhäusern der Basisversorgung, Schwerpunkt- und sonstigen Krankenhäusern nur minimale Unterschiede. Hingegen sind die Privatspitäler/Sanatorien Ausreißer mit langen Wartezeiten (siehe Abb. 2). Weiters stieg die Auslastung bei zehn Schmerzambulanzen in den letzten drei Jahren an. Diese liegt bei vier befragten Krankenhäusern zwischen 5 und 29 % und bei den restlichen sechs bei ca. 30 oder mehr Prozent.

### Schließungen von Schmerzambulanzen und deren Gründe

**Abb. 3:**
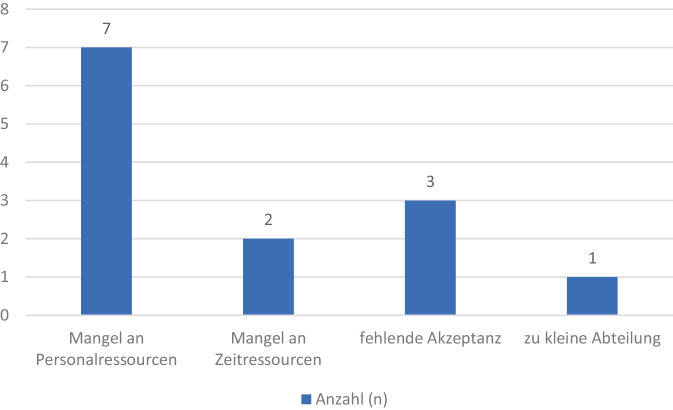
Begründungen für die Schließung von neun österreichischen Schmerzambulanzen in den Jahren vor 2023

Seit der letzten Umfrage über den Zustand der österreichischen Schmerzambulanzen im Jahr 2014 schlossen weitere neun Krankenhäuser ihre Schmerzambulanzen (siehe Tab. 1). Als Gründe hierfür wurden Einsparungen von Personalressourcen, Mangel an Zeitressourcen, fehlende Akzeptanz und eine zu kleine Abteilung beschrieben (siehe Abb. 3).

### Vollzeitbetrieb – Ist versus Soll

Basierend auf der geschätzten Anzahl an SchmerzpatientInnen wurde der Bedarf an „vollzeitbetriebenen“ Schmerzambulanzen errechnet (Die Rückmeldungen summativ gegengerechnet einem jährlichen Richtwert von 2400 PatientInnen einer vollzeitbetriebenen Schmerzambulanz mit einer wöchentlichen Betriebszeit von 40 Stunden.). Es zeigt sich, dass in Österreich 51,3 Schmerzambulanzen fehlen (siehe Tab. 2). Dies würde bedeuten, dass es für 70 % der chronischen SchmerzpatientInnen keine Versorgung durch eine Schmerzambulanz gibt (siehe Tab. 2).

**Tab. 2:** Schätzung chronischer Schmerzpatienten pro Bundesland

Bundesland	Wohnbevölkerung (Statistik Austria)^a^	Geschätzte Anzahl chronischer SchmerzpatientInnen^b^	Notwendige Schmerzambulanzen (bei 2400 Fällen pro Jahr)^c^	Vorhandene Schmerzambulanzen^d^	Vorhandene Schmerzambulanzen am Bedarf^d^
		Häufigkeit (*n*)	Häufigkeit (*n*)	Häufigkeit (*n*)	Prozent (%)
Wien	1.992.654	37.860	16	4,9	30,6
Niederösterreich	1.721.254	32.704	14	2,8	20,0
Oberösterreich	1.527.084	29.015	12	2,2	18,3
Steiermark	1.266.750	24.068	10	3,5	35,0
Tirol	773.491	14.696	6	2,9	48,3
Salzburg	569.870	10.828	5	1,8	36,0
Kärnten	568.919	10.809	5	2,6	52,0
Vorarlberg	408.343	7759	3	0,4	13,3
Burgenland	301.287	5724	2	0,5	25,0
Gesamt	9.129.652	173.463	73	21,7	29,7

^a^Daten der Bevölkerungsdichte der neun österreichischen Bundesländer (https://www.statistik.at/statistiken/bevoelkerung-und-soziales/bevoelkerung/bevoelkerungsstand/bevoelkerung-zu-jahres-/-quartalsanfangl)

^b^Schätzung der Häufigkeit chronisch schmerzerkrankter Patienten in Österreich; sehr hohe Chronifizierung (Richtwert 1,9 % pro 100.000 Einwohner; [7])

^c^Bei zwei fachärztlichen Schmerztherapeuten im Kernteam und 300 Fällen pro Quartal und fachärztlichen Schmerztherapeuten [15]

^d^Vorhandene Schmerzambulanzen basierend auf wöchentlichen Gesamtarbeitsstunden (40 h = 1 Schmerzambulanz)

### Personelle Besetzung und Therapieangebote für chronische PatientInnen

Eine gezielte Frage nach der Durchführung einer multimodalen Therapie (= konsensuelle Diagnostik und Therapie eines Teams aus mindestens ÄrztInnen, Pflegenden, PhysiotherapeutInnen, VertreterInnen der PSY-Disziplinen) bieten laut unseren Daten nur drei Krankenhäuser an. Details dazu und zur personellen Besetzung siehe Tab. 3.

**Tab. 3:** Überblick über Besetzung mit nichtärztlichem Personal in den Schmerzambulanzen verschiedener österreichischer Krankenhaustypen zum Zeitpunkt der Befragung (2022/23). Angaben in Werten pro Krankenhaus bzw. Prozent davon

	Universitätskliniken (%)	Schwerpunktkrankenhäuser (%)	Krankenhaus der Basisversorgung (%)	Privatklinik/Sanatorium (%)	^*^Sonstige Krankenhäuser (%)
DGKP in der Schmerzambulanz	5 (80)	18 (50)	14 (36)	1 (33)	2 (25)
Physio- und oder ErgotherapeutInnen in der Schmerzambulanz	1 (20)	7 (19)	7 (18)	k.A. (0)	k.A. (0)
PsychotherapeutInnen/klinische PsychologInnen in der Schmerzambulanz	k.A. (0)	2 (6)	2 (5)	k.A. (0)	k.A. (0)
Multimodale Schmerztherapie in Schmerzambulanzen	k.A. (0)	1 (3)	2 (5)	k.A. (0)	k.A. (0)

Angabe in Rückmeldungen je Krankenhaustyp, in Klammern in Prozent. k.A. = keine Angaben erhalten

**Abb. 4:**
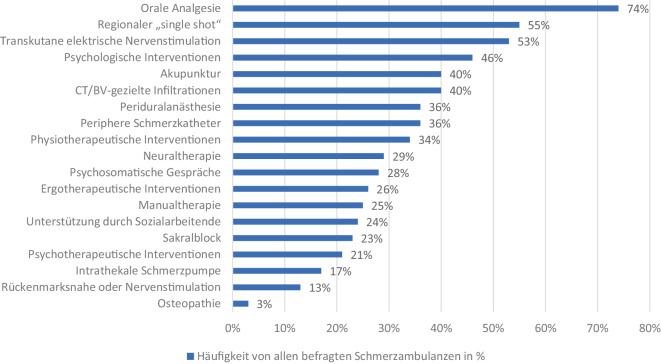
Die Häufigkeit der angebotenen Therapieoptionen von allen befragten Schmerzambulanzen in Österreich

Bei der Fragestellung nach einzelnen Therapieoptionen gaben 74 % der befragten Einrichtungen an, orale medikamentöse Therapie durchzuführen. Weiters sind invasive regionale Single-shot-Verfahren an zweiter Stelle mit 55 % (siehe Abb. 4). Diese Zahlen ergeben sich aus einer Mischung aus Schätzungen und intern geführten Statistiken der befragten Kliniken.

### Feedback der befragten ExpertInnen

**Abb. 5:**
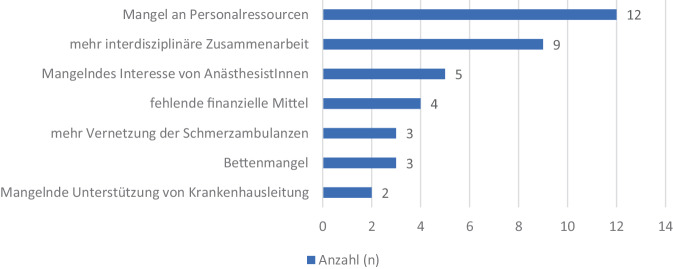
Auswertung der Kritikpunkte und des Feedbacks der befragten ExpertInnen rund um das Thema chronische Schmerztherapie

Im Freitext-Feedback wünschte sich die Mehrheit der TeilnehmerInnen mehr interdisziplinäre Zusammenarbeit und berichten von fehlenden Personalressourcen (siehe Abb. 5).

## Diskussion

Dies ist nach unserem Wissen die erste Publikation, die die aktuelle Entwicklung der Behandlung chronischer SchmerzpatientInnen in den österreichischen Schmerzambulanzen seit 2014 im Wandel der Zeit verfolgt und beschreibt.

### Existenz und Betrieb von Schmerzambulanzen

Laut unserer hier präsentierten Umfrage betreiben aktuell 51 von 96 teilnehmenden österreichischen Krankenhäusern eine Schmerzambulanz, davon jedoch nur mehr sieben im Vollbetrieb. Im Jahr 2014 existierten 44 Schmerzambulanzen mit 17,5 Ambulanzen im Vollbetrieb [21]. Nominell zeigt sich im Vergleich zu 2014 ein Plus an ca. 4 vollzeitbetriebenen Ambulanzen. Unter Berücksichtigung der an der Umfrage teilnehmenden Abteilungen bedeutet es umgekehrt ein Minus von 7,5 % an Schmerzambulanzen in 2023. Aktuell fehlen damit in Österreich 51,3 Schmerzambulanzen, mehr als 2014.

Natürlich kann infrage gestellt werden, welche Form an multimodaler Schmerztherapie die mehrfach beschriebenen 20 % der chronischen SchmerzpatientInnen benötigen: Muss es eine intrahospitale Schmerzambulanz sein oder kann dies im niedergelassenen Bereich z. B. über die AllgemeinmedizinerInnen oder FachärztInnen organisiert werden? Eine Evaluierung über die Notwendigkeit der Einbindung in eine multimodale Schmerzambulanz ist hierfür dringend notwendig, sodass PatientInnen mit Tumorschmerzen oder orthopädischen Krankheitsbildern zuerst anderen Therapieformen wie palliativer Therapie bzw einer kausalen Behandlung zugeführt werden können [22].

### Verteilung der Schmerzambulanzen

In der Bundesländerverteilung führte 2014 die Steiermark mit zehn Schmerzambulanzen. Aktuell findet man in Wien mit zwölf Ambulanzen die meisten Schmerzambulanzen [21]. Dies bedeutet aktuell einen nominellen Zuwachs von drei Ambulanzen in Wien, von zwei Ambulanzen in Oberösterreich und Vorarlberg und von einer Ambulanz in Kärnten, Tirol und Burgenland. Ohne Berücksichtigung der Öffnungszeiten bedeutet dies einen scheinbaren Fortschritt in der Versorgung chronischer SchmerzpatientInnen.

### Betrieb und Betriebszeiten von Schmerzambulanzen

Eine Reduktion der Öffnungszeiten berichteten zehn der teilnehmenden 51 Ambulanzen, zum Großteil mit einer Reduktion von mehr als 50 % ihrer bisherigen Öffnungszeiten. Dies ist vergleichbar zur Umfrage von 2014 [21].

Unsere aktuellen Daten zeigen eine durchschnittliche Wartezeit von 27 bzw. 28 Tagen in Basis- und Schwerpunktkrankenhäusern für PatientInnen mit chronischen Schmerzen. Die mehr als doppelt so langen Wartezeiten von Privatspitälern/Sanatorien sind unserer Ansicht nach vernachlässigt, da sie mit einer Anzahl von zwei aktiven Schmerzambulanzen in Österreich nicht repräsentativ für eine flächendeckende Versorgung sind. Verglichen mit sonstigen Wartezeiten im Gesundheitswesen im europäischen Raum sind diese noch kurz, da es laut einer Umfrage 2023 zwischen 28 und 77 Tage dauert, um einen Ersttermin zu bekommen [10]. Eine Studie von Treede et al. aus Kanada berichtet dies ebenso. Nur ein Drittel der befragten PatientInnen bekam innerhalb von drei Monaten einen Termin [22]. Ein Trend in Richtung Wartezeitenverlängerung zeigt sich in einer Studie von Lynch et al. PatientInnen sind in 65 % der befragten Kliniken seit der COVID-19-Pandemie längeren Wartezeiten ausgesetzt. Eine exakte Dauer in Tagen wurde nicht erhoben. Als Folge daraus registrierten 76 % der von ihnen befragten Kliniken relevant höhere Schmerzniveaus bei ihren chronischen SchmerzpatientInnen. Zusätzlich berichteten 71 % der Kliniken von einem erschwerten Behandlungszugang für die ohnehin schon eingeschränkt mobilen SchmerzpatientInnen [16].

### Schließungen von Schmerzambulanzen und deren Gründe

Seit 2014 haben neun österreichische Schmerzambulanzen gänzlich geschlossen, begründet wird dies vorrangig durch die mangelnden personellen Ressourcen. Allerdings lassen die Antworten weiterführend auf ein vielschichtigeres Problem rückschließen, wie fehlendes Interesse und mangelnde Finanzierung [21].

### Vollzeitbetrieb – Ist versus Soll

Im Vergleich zu unserer Umfrage von 2014 zeigt sich ein Plus an 4,2 vollzeitbetriebenen Schmerzambulanzen, jedoch beim vorbestehenden Defizit im Vergleich von damals 49,5 und aktuell 51,3 Ambulanzen eine tendenzielle Verschlechterung in der Versorgung von chronischen SchmerzpatientInnen [21]. Bei einem gesundheitsökonomisch kalkulierten Bedarf von aktuell 51,3 Vollzeitambulanzen bedeutet dies eine fehlende Versorgung für gut 70 % der Bevölkerung. Mit einem Blick auf die Entwicklung in den Bundesländern zwischen den beiden Befragungen weisen alle bis auf einzig Vorarlberg ein Defizit auf [13].

### Personelle Besetzung der Schmerzambulanzen

Multimodale Therapie benötigt die Präsenz von VertreterInnen zumindest der Ärzteschaft, Pflege, Physiotherapie und der Psy-Disziplinen im Team der Schmerzambulanz. In Österreich bleibt dies jedoch primär ein Wunsch, wie unsere aktuellen Zahlen bestätigen: Mit absteigender Wahrscheinlichkeit gehört eine Pflegeperson ins Team, die anderen Berufsgruppen sind Raritäten. Unklar ist, ob diese Berufsgruppen primär anderen Arbeitsbereichen zugeordnet sind und in der Schmerzambulanz ohne direkte Zuordnung unterstützen [18].

### Therapieangebote für chronische SchmerzpatientInnen

Das nur drei der befragten Krankenhäuser (Schwerpunkt- und Basisversorgung) laut Überblicksfrage „multimodale Schmerztherapie“ anbieten, zeigt wie vernachlässigt die Versorgung chronifizierter SchmerzpatientInnen ist.

Der Blick auf die nachfolgende Detailfrage nach den verwendeten Therapieformen bestätigt dies leider. Die Teilnehmenden beschreiben nachvollziehbar die orale medikamentöse Therapie als häufigste Aktivität, gefolgt von der invasiven Single-shot-Regionalanästhesie und der transkutanen elektrischen Nervenstimulation. Die aktuellen Daten zeigten eine klare Priorisierung invasiver Methoden vor multimodalen Angeboten. Ähnliche Zahlen beschrieben Fidahic et al. im Jahr 2013 als Ergebnis einer Online-Umfrage in 21 kroatischen Kliniken (Rücklaufquote von 95 %): Orale medikamentöse Einstellungen kamen in allen befragten Schmerzkliniken zum Einsatz, gefolgt von der transkutanen elektrischen Nervenstimulation mit 90 % und Akupunktur mit 81 %. Multimodale Konzepte mit Einbindung der Physio‑, Ergo- und Psychotherapie wurden nicht angeboten [9].

Es zeigt sich eine bedenkliche Häufigkeit von Regionalanästhesien bei 55 % der befragten Kliniken, was eine hohe Zahl an invasiven Interventionen darstellt. In der multimodalen Schmerztherapie werden diese Verfahren stets zurückhaltend und unter strenger Indikationsstellung durchgeführt. Es ist zu befürchten, dass durch die hohe Anzahl dieser Interventionen – ohne Einbindung in ein multimodales Therapiekonzept – die Chronifizierung der SchmerzpatientInnen verstärkt werden könnte. Um dies zu verhindern, wäre es im Sinne der PatientInnen, invasive Verfahren nur im Sinne der multimodalen Schmerztherapie oder nach Einholung einer „second opinion“ einzusetzen.

Eine moderne multimodale, interdisziplinäre Schmerztherapie baut auf einer ausgewogenen Mischung aus primär konservativen ärztlichen, physio- und psychotherapeutischen Maßnahmen auf. Die beiden letzteren wurden in unserer Umfrage deutlich seltener genannt, was die leitlinienkonforme Behandlungsqualität von chronischen SchmerzpatientInnen in Österreich infrage stellt. Ähnliche Probleme publizierte bereits 2012 eine Studie über den Status der Schmerzambulanzen in Deutschland: Die befragten Klinken gaben an, dass in nur 44 % der Schmerzambulanzen PsychologInnen eingebunden wurden [8].

Als potenzielle Ursache dafür wird in unseren Daten auf den hohen Bedarf an besseren Rahmenbedingungen für die Zusammenarbeit von verschiedenen medizinischen Berufsgruppen in vielen Schmerzambulanzen hingewiesen: Der aktuelle Personalmangel und die hohen Personalkosten, aggraviert durch die fehlende Wertschätzung der Leistung der SchmerztherapeutInnen im fachärztlichen Team, sind bestehende Probleme seit der letzten Umfrage von 2014 [21].

### Feedback der ExpertInnen

Um die von den Befragten bemängelten Umstände zu verbessern, müsste ein Umdenken und daraus abgeleitet ein großer Eingriff in die aktuelle Entwicklung und Struktur unseres Gesundheitssystems stattfinden. Schmerztherapie stellt mit einem Blick auf das aktuelle Honorierungssystem einen Negativposten dar.

Die COVID-Pandemie verschärfte nachhaltig die schon angespannte personelle Situation in den Spitälern. Aus Sicht der Führungsebene erfordert dies eine Konzentration der Ressourcen auf das Kerngeschäft sowie eine Verkürzung der Belegstage. Dies führt dazu, dass einerseits Personal aus den Schmerzambulanzen abgezogen wird und andererseits die PatientInnen entweder mit einer nicht funktionierenden oralen Schmerzmedikation (meist zu späte Umstellung von systemisch auf oral) oder in komplexen Situationen ohne Schmerzkonsil (bei Personalmangel in der Schmerzambulanz) entlassen werden [6]. Zusätzlich fehlen damit Möglichkeiten zur Nachbehandlung protrahierter poststationärer Schmerzen (wie z. B. im Rahmen von Wundheilungsstörungen oder neu aufgetretenen neuropathischen Schmerzen) oder chronischer SchmerzpatientInnen. Wenn gleichzeitig die Wertschätzung im Abteilungsteam für das Schmerzteam verloren geht, reduziert dies nachhaltig die Arbeitszufriedenheit und Behandlungsqualität und erhöht die Abwanderungsrate aus dem Spitalsbereich [20].

Neben finanziellen Ressourcen für die Krankenhäuser wäre es notwendig, vermehrt auf präventive und gesundheitsfördernde Elemente wie beispielsweise die Patientenedukation zu setzen. Exemplarisch wäre das dänische Modell für PatientInnen, die auf eine Knieprothesenoperation warten (GLA:D), zu nennen. In diesem Programm werden präoperative Patientenedukation und Gruppenphysiotherapie genutzt, um die Beschwerden sowie Operationen bei diesen PatientInnen hochrelevant zu reduzieren [11].

### Limitationen

Wir verwendeten einen starren Fragebogen, der eine rasche Beantwortung und hohe Rücklaufrate sicherte, jedoch keine Möglichkeit der Nachfrage ließ. Obwohl diese Umfrage auf jener aus dem Jahr 2014 aufbaute, war es nötig, die Fragen zur Präzisierung nachhaltig zu adaptieren. Ein direkter statistischer Vergleich war erhofft, aber nicht möglich. Wir führten unsere Umfrage rein online durch, was ebenfalls die Möglichkeit der Nachfrage reduzierte. Der gesamte Fragebogen war weder validiert noch auf „interrater reliability“ geprüft. Mögliche Missverständnisse bei der Beantwortung der Fragen könnten dadurch entstanden sein. Nicht alle Krankenhäuser in Österreich antworteten, jedoch urgierten die AutorInnen alle jene Krankenhäuser, in denen die Existenz einer Schmerzambulanz bekannt war. Dort war der Rücklauf vollständig, was uns die hohe Relevanz der erhobenen Daten und eine repräsentative Aussage sichert.

Es ist kritisch zu hinterfragen, ob von den laut Bericht der ÖSG 2020 etwa 20 % der österreichischen Bevölkerung mit chronischen Schmerzen wirklich alle eine Schmerzambulanz oder eine tagesklinische/stationäre multimodale schmerztherapeutische Behandlung benötigen. Eine Klassifizierung von diesen PatientInnen wäre notwendig, um zu selektieren, ob ein einmaliger Kontakt in der Schmerzambulanz bzw. eine rein ambulante Versorgung im niedergelassenen Bereich ausreichend oder eine tatsächliche höherfrequente Einbindung in einer multimodalen Schmerzambulanz notwendig ist.

Die Ergebnisse zur multimodalen Schmerztherapie lassen die Frage aufkommen, ob es ein Missverständnis bei der Beantwortung des Fragebogens gab. Es irritiert, dass keine der befragten Universitätsklinken multimodale Schmerztherapie in ihren Schmerzambulanzen betreibt. Uns liegen jedoch Daten aus Umfragen mit demselben Fragebogen in Deutschland vor, die klar den Einsatz der multimodalen Therapie favorisieren und bestätigen [24]. Kritisch kann die häufige Abwesenheit von interdisziplinärer Zusammenarbeit bei den befragten ExpertInnen hinterfragt werden, da dies aufzeigt, dass das Konzept von multimodaler Schmerztherapie kaum in der Praxis der befragten Kliniken angewandt wird.

## Ausblick

Unsere Ergebnisse bestätigen die aktuelle Tendenz zur sich fortsetzenden Reduktionen der Existenz, Öffnungszeiten bzw. schmerztherapeutischen Angebote in Österreich. Gut 70 % der österreichischen schmerzgeplagten Bevölkerung fehlt die Möglichkeit zur zeitgerechten und leitlinienkonformen Behandlung. Invasive Verfahren werden häufiger angeboten als die multimodale Therapie, die eigentliche Kernaufgabe der interdisziplinären Schmerzambulanzen. Fehlende personelle und räumliche Ressourcen bei gleichzeitig abnehmender Wertschätzung im intrahospitalen Setting beschleunigen diese negative Entwicklung. Mit einer spürbaren Verschlechterung der Behandlungsqualität, des Wettbewerbsvorteils sowie der direkten, aber vor allem indirekten Gesundheitskosten (Krankenstände, Krankentage, Berufsausfälle) muss deswegen gerechnet werden. Politik, die Krankenkassen, die Krankenanstaltenträger sowie die Krankenhausführung sind jetzt gefordert, diesen höchst bedenklichen Trend zu stoppen und rasch Gegenmaßnahmen einzuleiten.

## Fazit für die Praxis

- Trotz einer nominellen Zunahme an Schmerzambulanzen sank die summative Anzahl der Betriebsstunden relevant seit der Letztbefragung 2014.
- Die Anzahl der vollzeitbetriebenen Schmerzambulanzen hat sich seit 2014 fast halbiert.
- Multimodale Schmerztherapie kommt nur in wenigen Kliniken zur Anwendung.
- Bedenklich hoher Anteil an invasiven Interventionen in Österreichs Schmerzambulanzen
- Indirekte Gesundheitskosten könnten als Reaktion auf die schlechte Qualität der Versorgung steigen.

## Data Availability

Die Daten, die die Ergebnisse dieser Publikation stützen, sind auf begründete Anfrage beim korrespondierenden Autor erhältlich.
